# Supplementary material for: Sirtuin 6 maintains epithelial STAT6 activity to support intestinal tuft cell development and type 2 immunity
Source: Nat Commun. 2022 Sep 3;13:5192. doi: 10.1038/s41467-022-32846-4 (PMC9440928; doi:10.1038/s41467-022-32846-4)
Supplement: Supplementary file 1 — Supplementary Information [file 41467_2022_32846_MOESM1_ESM.pdf]

Supplementary Information

Sirtuin 6 maintains epithelial STAT6 activity to support intestinal tuft cell development and type 2 immunity

Xiwen Xiong*, Chenyan Yang, Wei-Qi He, Jiahui Yu, Yue Xin, Xinge Zhang, Rong Huang, Honghui Ma, Shaofang Xu, Zun Li, Jie Ma, Lin Xu, Qunyi Wang, Kaiqun Ren, Xiaoli S. Wu, Christopher R. Vakoc, Jiateng Zhong, Genshen Zhong, Xiaofei Zhu, Yu Song, Hai-Bin Ruan, Qingzhi Wang*

*To whom correspondence should be addressed.

Email: xwxiong@xxmu.edu.cn (Xiwen Xiong), wqzmgl@126.com (Qingzhi Wang)

Supplementary Figure 1

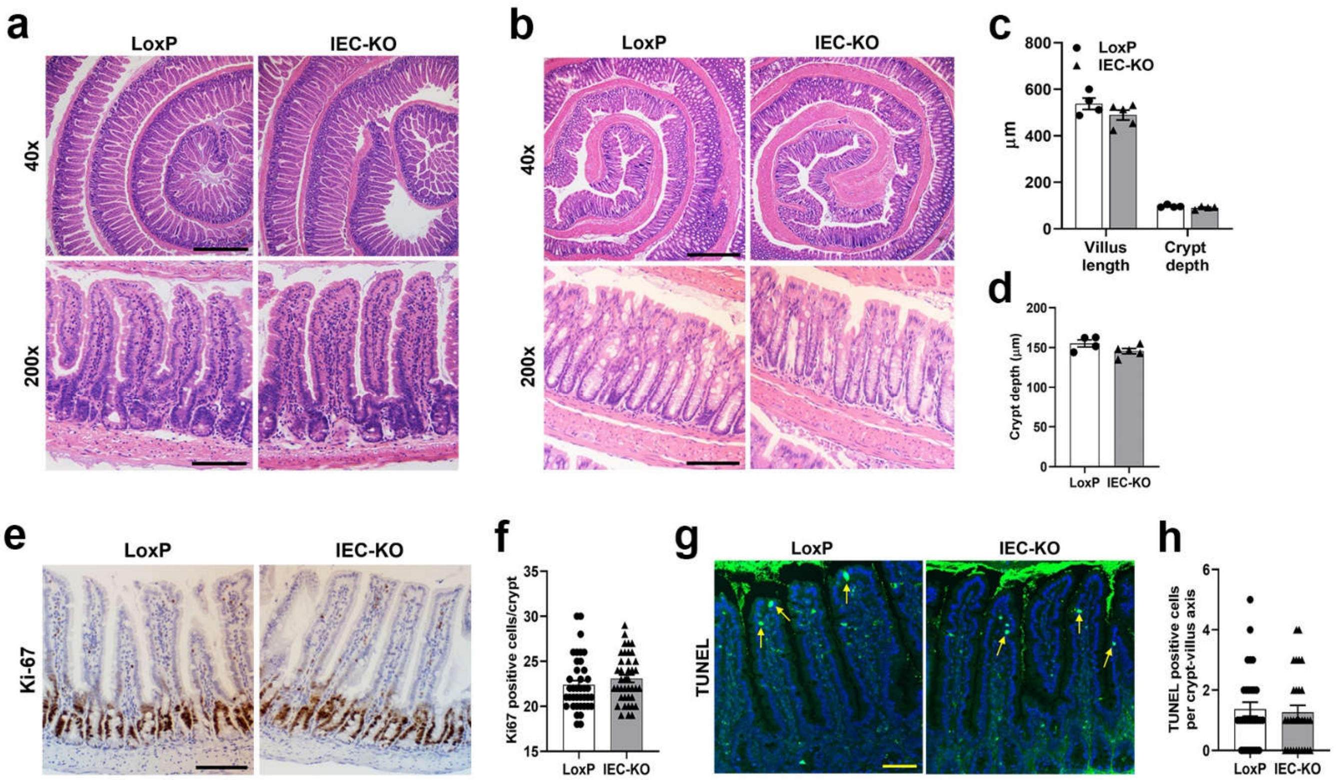

Supplementary Fig. 1. IEC-KO mice do not display abnormalities in intestinal mucosal architecture. 2-3-month-old male LoxP and IEC-KO mice were subjected to the following assays. **(a, b)** Representative H&E images of jejunum (a) and colon (b). **(c)** Analyses of the jejunal villus length and crypt depth. n=4 (LoxP) and 5 (IEC-KO) mice for villus length, n=4 mice/group for crypt depth; 30 villi or 20 crypts counted for each mouse. **(d)** Analysis of the colonic crypt depth. n=4 (LoxP) and 5 (IEC-KO) mice; 30 crypts counted for each mouse. **(e)** Immunostaining of Ki67 in the jejunum. **(f)** Quantification of Ki67 positive cells shown in (e). n=40 crypts/group. **(g)** TUNEL staining in the jejunum with arrows denoting positive nuclei. **(h)** Quantification of TUNEL positive cells shown in (g). n=30 crypt-villus units/group. Data are presented as mean ± SEM. Statistical analyses were carried out using two-tailed unpaired *t* test. Scale bars, 500μm in (a, b, upper), 100μm in (a, b, lower and e); 50μm in (g). Source data are provided as a Source Data file.

Supplementary Figure 2

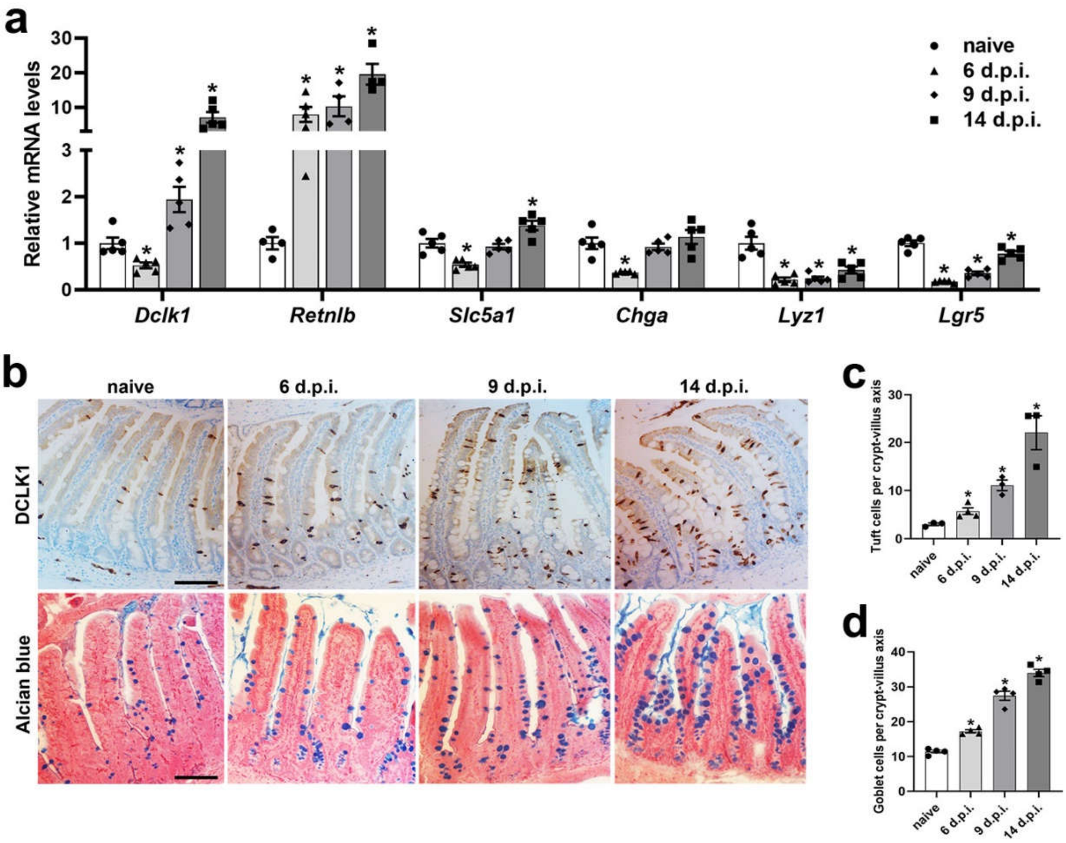

Supplementary Fig.2. *H.poly* infection leads to hyperplasia of intestinal tuft and goblet cells in mice. 2-month-old C57BL/6J male mice were infected with *H.poly* and analyzed on indicated days post-infection. **(a)** qPCR analysis of IEC cell markers expression in the jejunal IECs. n=5 mice/group; *Dclk1*, *p*=0.0151 (6 dpi), 0.022 (9 dpi), 0.0171 (14 dpi); *Retnlb*, *p*=0.0328 (6 dpi), 0.0481 (9 dpi), 0.0086 (14 dpi); *Slc5a1*, *p*=0.0045 (6 dpi), 0.0023 (14 dpi); *Chga*, *p*=0.0066 (6 dpi); *Lyz1*, *p*=0.0032 (6 dpi), 0.0039 (9 dpi), 0.0101 (14 dpi); *Lgr5*, *p*=0.0001 (6 dpi), <0.0001 (9 dpi), 0.026 (14 dpi); **(b)** Tuft and goblet cells were examined by DCLK1 immunostaining and Alcian blue staining, respectively in the jejunum (200X). **(c, d)** Quantification of tuft and goblet cells shown in (b). n=3, 4, 3, 3 mice/group, respectively in (c), n=4 mice/group in (d); 40 (c) and 20 (d) crypt-villus units counted for each mouse; *p*=0.0224 (6 dpi), 0.014(9 dpi), 0.0329 (14 dpi) in (c); *p*<0.0001 (6 dpi), 0.0005 (9 dpi), <0.0001 (14 dpi) in (d). Data are presented as mean ± SEM. All *p* values were generated by two-tailed unpaired *t* test. *p<0.05 vs naive. Scale bars, 100µm. Source data are provided as a Source Data file.

Supplementary Figure 3

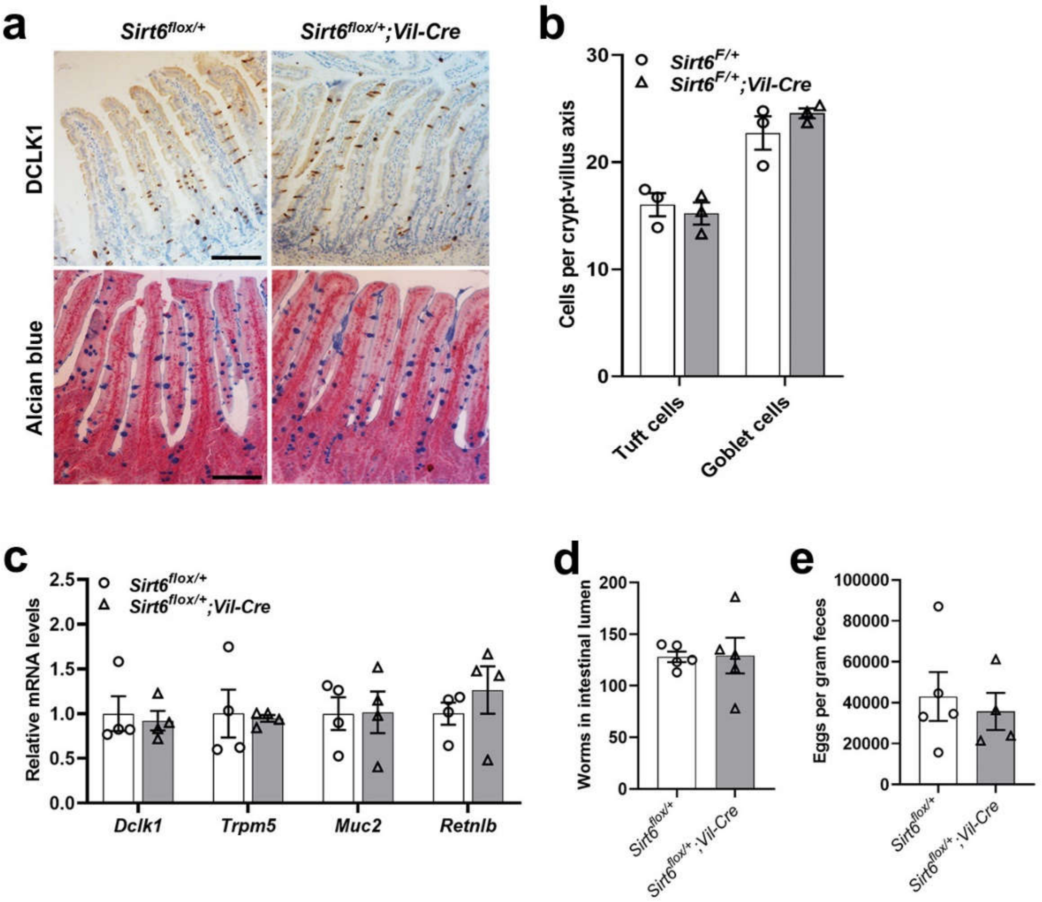

Supplementary Fig.3. *Sirt6$^{flox/+}$;Vil-Cre* mice exhibit normal anti-helminth responses. 2-month-old male *Sirt6$^{flox/+}$* and *Sirt6$^{flox/+}$;Vil-Cre* mice were infected with *H.poly* and analyzed on day 14 post-infection. **(a)** Tuft and goblet cells were examined by DCLK1 immunostaining and Alcian blue staining, respectively in the jejunum (200X). **(b)** Quantification of tuft cells and goblet cells shown in (a). n=3 mice/group;15 crypt-villus units counted for each mouse. **(c)** qPCR analysis of tuft and goblet cell markers expression in the jejunal IECs. n=4 mice/group. **(d, e)** Analysis of parasite burden by quantification of adult worms in intestinal lumen (d) and eggs in feces (e). n=5 mice/group in (d), n=5 (*Sirt6$^{flox/+}$*) and 4 (*Sirt6$^{flox/+}$*;Vil-Cre) mice in (e). Data are presented as mean ± SEM. Statistical analyses were carried out using two-tailed unpaired *t* test. Scale bars, 100μm. Source data are provided as a Source Data file.

Supplementary Figure 4

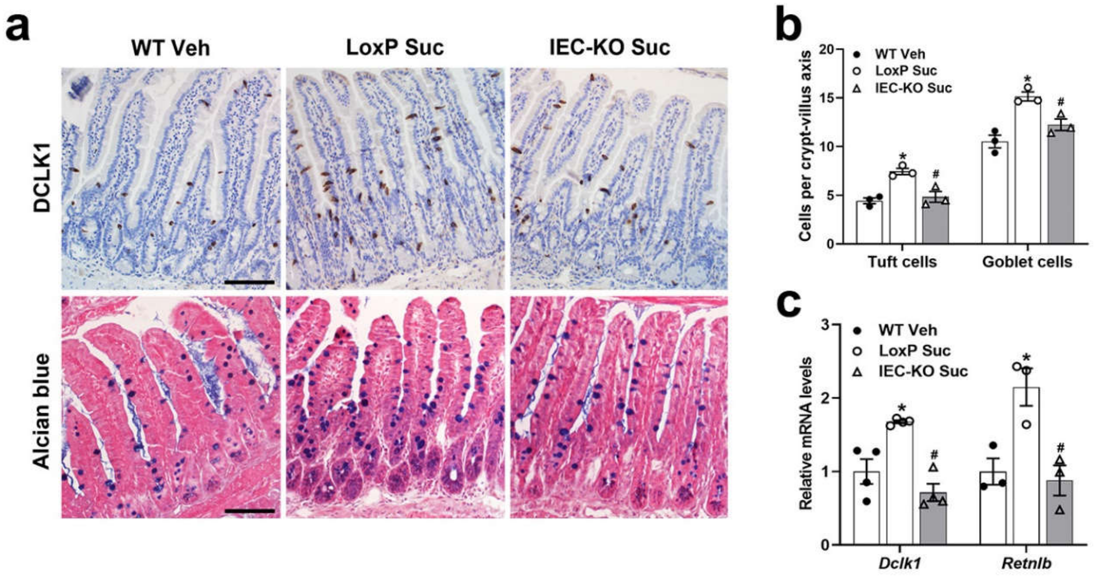

Supplementary Fig.4. Deletion of *Sirt6* in IECs leads to impaired succinate-induced epithelial responses. 4-month-old male LoxP and IEC-KO mice were given 150mM succinate and WT mice were given control water for 7 days. **(a)** Tuft and goblet cells were examined by DCLK1 immunostaining and Alcian blue staining, respectively in the jejunum (200X). **(b)** Quantification of tuft cells and goblet cells shown in (a). n=3 mice/group; 15 crypt-villus units counted for each mouse; WT Veh vs LoxP Suc, p=0.0024 (tuft cell), 0.0059 (goblet cell); LoxP Suc vs IEC-KO Suc, p=0.024 (tuft cell), 0.02 (goblet cell). **(c)** qPCR analysis of tuft and goblet cell markers expression in the jejunal IECs. n=4 (*Dclk1*) and 3 (*Retnlb*) mice/group; WT Veh vs LoxP Suc, p=0.0267 (*Dclk1*), 0.0253 (*Retnlb*); LoxP Suc vs IEC-KO Suc, p=0.0031 (*Dclk1*), 0.0193 (*Retnlb*). Data are presented as mean ± SEM. All p values were generated by two-tailed unpaired t test. *p<0.05 vs WT Veh, #p<0.05 vs LoxP Suc. Scale bars, 100μm. Source data are provided as a Source Data file.

Supplementary Figure 5

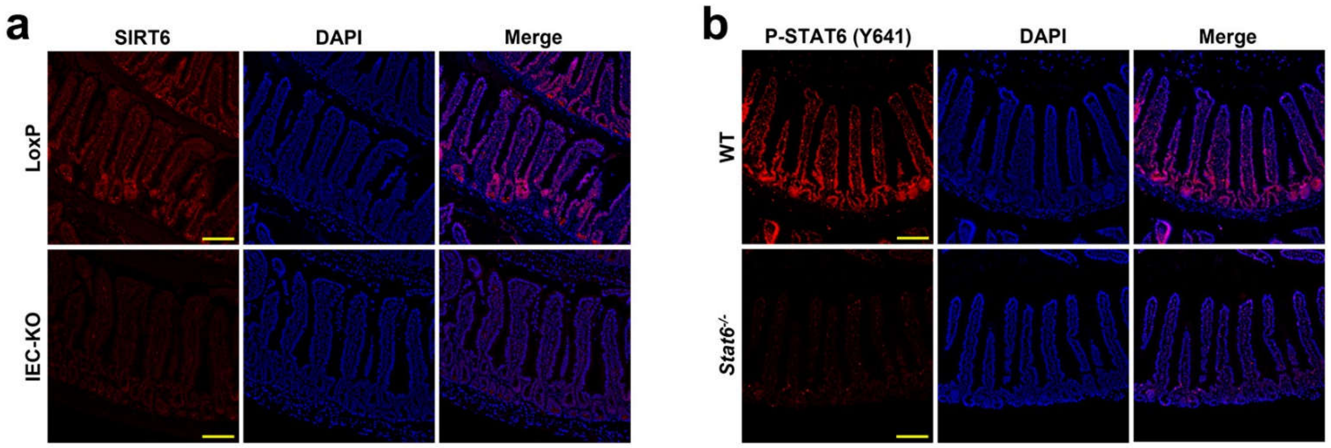

Supplementary Fig.5. Validation of specificity of anti-SIRT6 and anti-P-STAT6 (Y641) by immunostaining. (a) Immunostaining for SIRT6 in the jejunum of LoxP and IEC-KO mice. **(b)** Immunostaining for P-STAT6 (Y641) in the jejunum of WT and *Stat6*$^{-/-}$ mice. Scale bars, 100μm. Experiments were repeated two times for (a) and (b).

Supplementary Figure 6

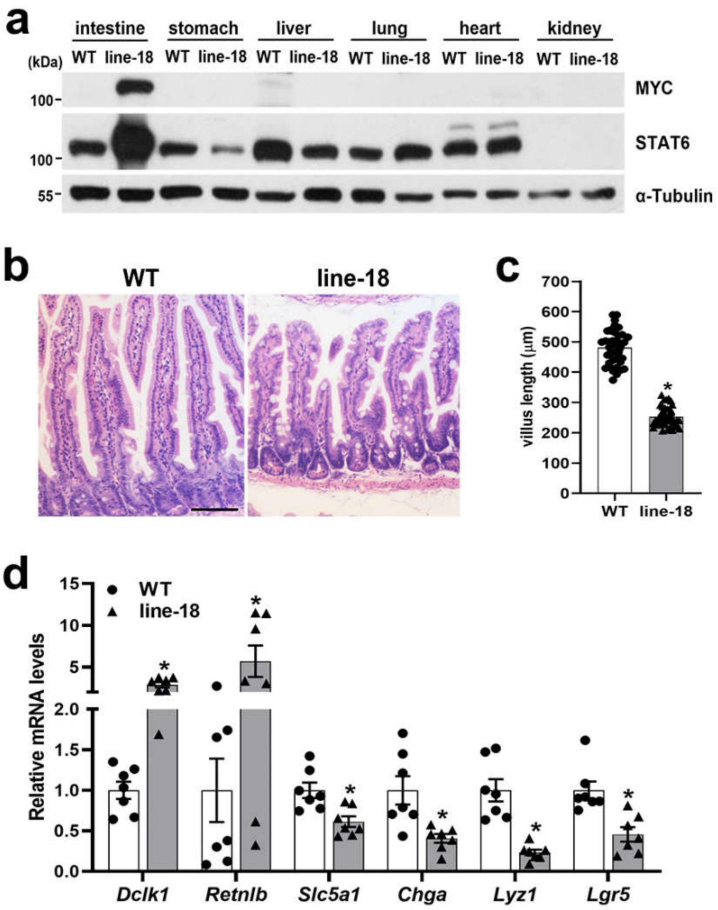

Supplementary Fig.6. Phenotypic characterization of TgSTAT6vt-line18 mice. 2-month-old male WT and line-18 mice were subjected to the following analyses. **(a)** Western blot analysis of transgene STAT6vt expression in different tissues. **(b)** Representative H&E images of the jejunum. **(c)** Analysis of the jejunal villus length. n=40 villi/group; $p<0.0001$. **(d)** qPCR analysis of IEC cell markers expression in the jejunal IECs. n=7 mice/group; $p=0.0007$ (*Dclk1*), 0.0476 (*Retnlb*), 0.0067 (*Slc5a1*), 0.0138 (*Chga*), 0.0011 (*Lyz1*), 0.0025 (*Lgr5*). Data are presented as mean ± SEM. All *p* values were generated by two-tailed unpaired *t* test. *$p<0.05$. Scale bars, 100μm. Source data are provided as a Source Data file.

Supplementary Figure 7

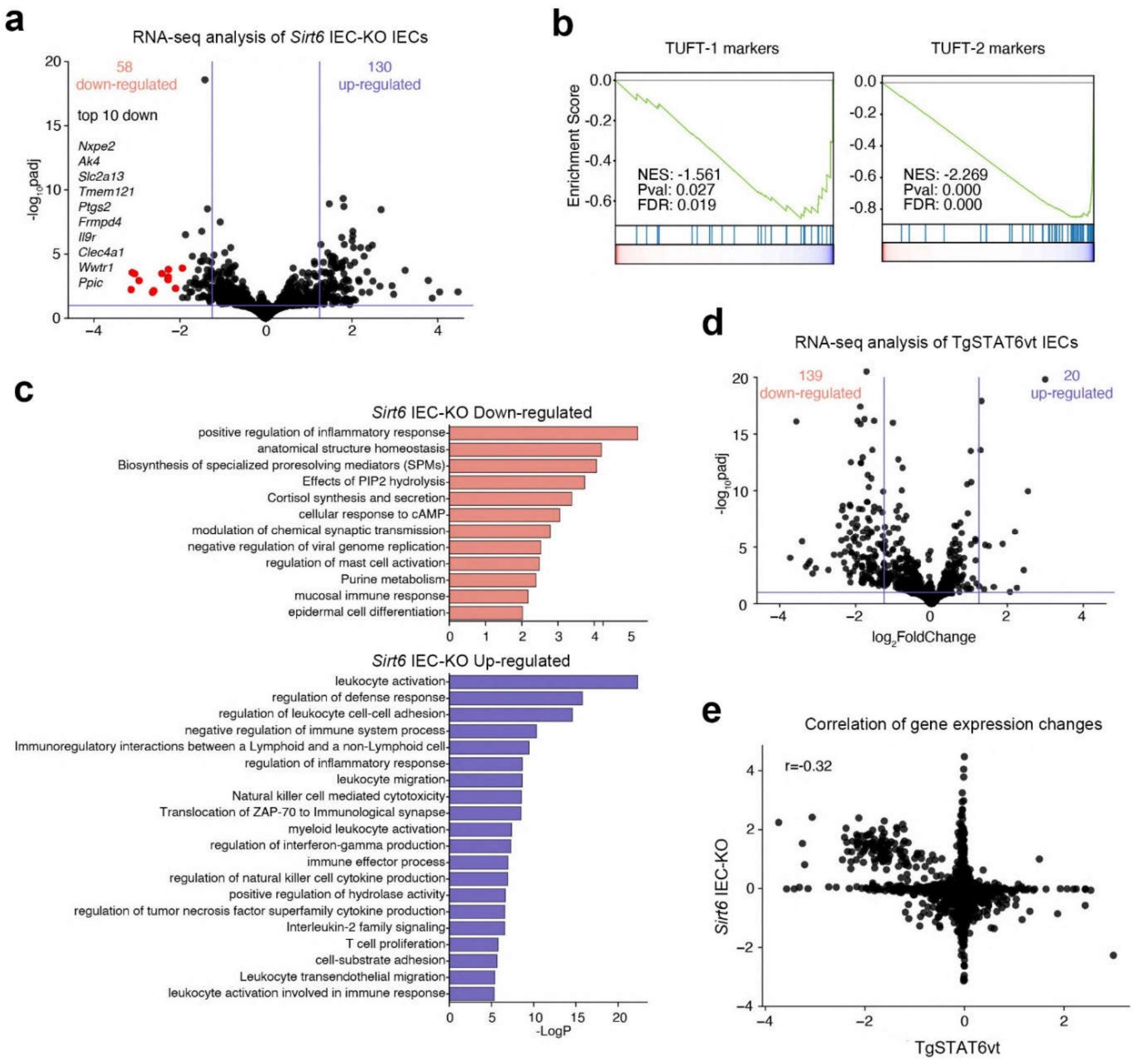

Supplementary Fig.7. RNA-seq analysis reveals that epithelial deletion of *Sirt6* reduces the expression of tuft cell identity genes. 2-month-old male LoxP&IEC-KO mice and WT&TgSTAT6vt mice were subjected to IECs isolation and RNA-seq analysis. **(a)** A volcano plot illustrating differentially expressed gene (log2(fold-change) from DESeq2) in IECs from IEC-KO mice versus LoxP mice, with top 10 down-regulated genes highlighted in red. n=3 mice/group. **(b)** Gene set enrichment analysis (GSEA) of downregulated genes (n=58) in IECs from IEC-KO versus LoxP mice with respect to tuft-1 and tuft-2 marker genes defined by Harber *et al.* (Nature. 551(7680):333-339). **(c)** Ontology analysis of the 58 significantly down-regulated and 130 upregulated genes in IECs from IEC-KO mice using Metascape. GO terms are ranked by their significance (Benjamini-Hochberg corrected *p* value). **(d)** A volcano plot illustrating differentially expressed genes (log2(fold-change) from DESeq2) in IECs from TgSTAT6vt versus WT mice. n=3 mice/group. **(e)** RNA-seq comparison analysis. Scatterplot depicts the log2(fold-change) (from DESeqe2) of IEC-KO versus LoxP or TgSTAT6vt versus WT.

Supplementary Figure 8

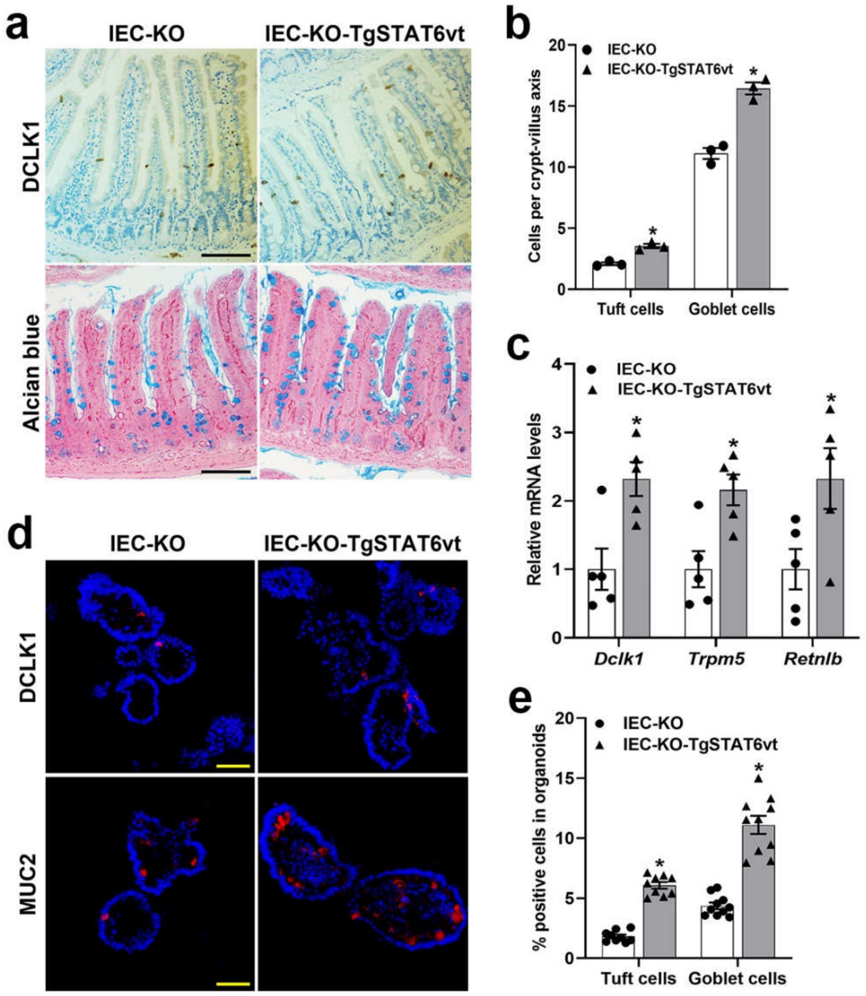

Supplementary Fig.8. Ectopic expression of STAT6vt in the intestinal epithelium promotes tuft and goblet cell differentiation in IEC-KO mice. 2-3-month-old male IEC-KO and IEC-KO-TgSTAT6vt mice were subjected to the following assays. **(a)** Tuft and goblet cells were examined by DCLK1 immunostaining and Alcian blue staining, respectively in the jejunum (200X). **(b)** Quantification of tuft and goblet cells shown in (a). n=3 mice/group; 50 crypt-villus units counted for each mouse; p=0.0042 (tuft cell), 0.0014 (goblet cell). **(c)** qPCR analysis of *Dclk1*, *Trpm5* and *Retnlb* expression in the jejunal IECs. n=5 mice/group; p=0.0101 (*Dclk1*), 0.0103 (*Trpm5*), 0.043 (*Retnlb*). **(d)** Tuft and goblet cells were labelled by anti-DCLK1 and anti-Muc2, respectively, in frozen sections of organoids (200X). **(e)** Quantification of tuft and goblet cells shown in (d). n=9 (tuft cell) and 10 (goblet cell) organoids/group; p<0.0001 (tuft, goblet cell). Data are presented as mean ± SEM. All p values were generated by two-tailed unpaired t test. *p<0.05. Scale bars, 100μm in (a); 50μm in (d). Source data are provided as a Source Data file.

Supplementary Figure 9

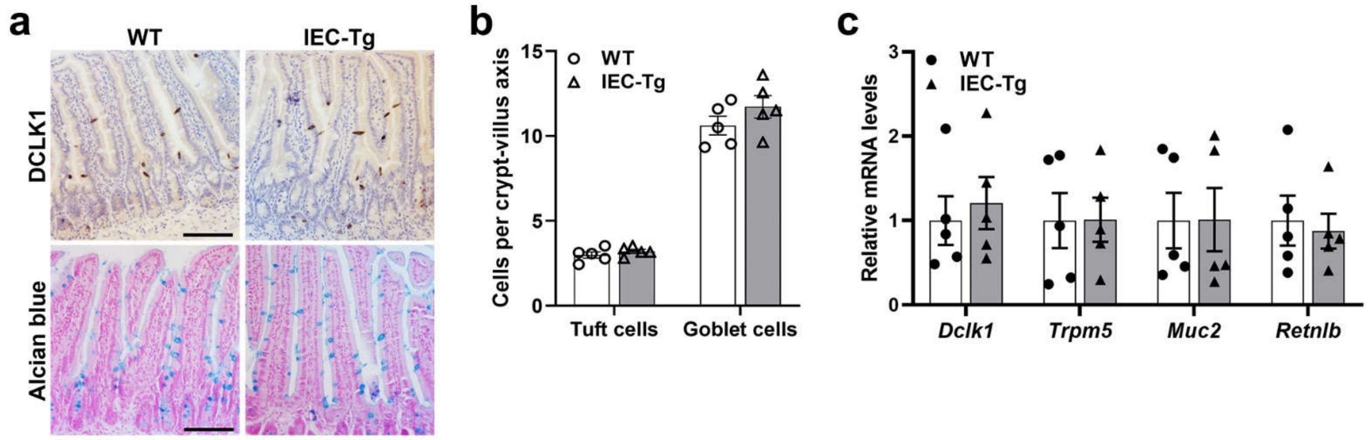

Supplementary Fig.9. IEC-Tg and WT mice exhibit comparable tuft and goblet cell abundance in the naïve state. 2-month-old male WT and IEC-Tg mice were subjected to following analyses. **(a)** Tuft and goblet cells were examined by DCLK1 immunostaining and Alcian blue staining, respectively in the jejunum (200X). **(b)** Quantification of tuft and goblet cells shown in (a). n=5 mice/group; 30 crypt-villus units counted for each mouse. (c) qPCR analysis of tuft and goblet cell markers expression in the jejunal IECs. n=5 mice/group. Data are presented as mean ± SEM. Statistical analyses were carried out using two-tailed unpaired *t* test. Scale bars, 100µm. Source data are provided as a Source Data file.

Supplementary Figure 10

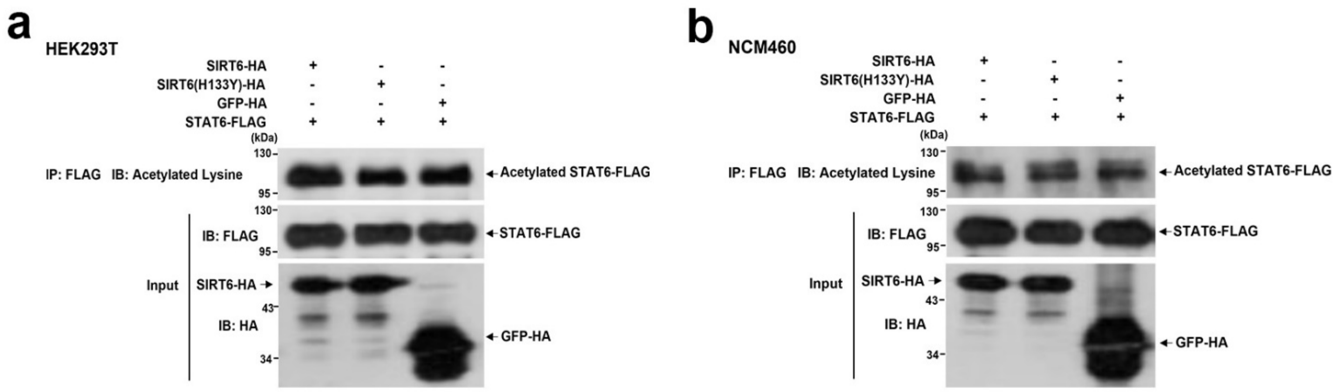

Supplementary Fig.10. SIRT6 does not influence the acetylation status of STAT6.

(a, b) Effects of SIRT6 overexpression on STAT6 acetylation. STAT6-FLAG was immunoprecipitated from HEK293T cells (a) or NCM460 cells (b) transfected with SIRT6-HA or GFP-HA and blotted for total acetylation. Source data are provided as a Source Data file. Experiments were repeated two times for (a) and (b).

Supplementary Figure 11

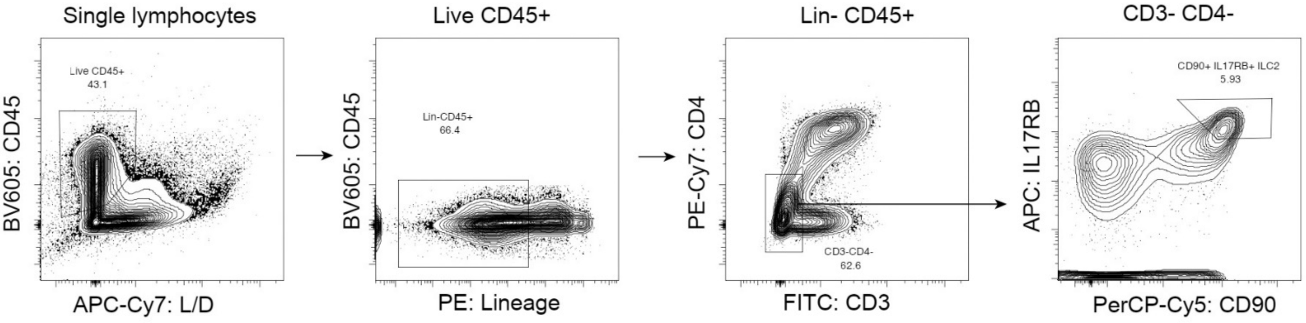

Supplementary Fig.11. Gating strategies and representative flow cytometry plots to detect ILC2s.

Supplementary Table 1. The sequence information of the primers used in this study

Name	Species	Application	Forward (5'-3')	Reverse (5'-3')
Chga	mouse	RT-qPCR	ATGACAAAAGGGGACACCAA	GTCTCCAGACACTCAGGGCT
Dclk1	mouse	RT-qPCR	TGAAGCGCCTGTACACTCTG	CTTCTCTGGTCCACATGCAA
Il13	mouse	RT-qPCR	TGTGTCTCTCCCTCTGACCC	CACACTCCATACCATGCTGC
Il25	mouse	RT-qPCR	AGCAGGGCCATCTCTCCT	GTCTGTAGGCTGACGCAGTG
Il4	mouse	RT-qPCR	TGAACGAGGTCACAGGAGAA	CGAGCTCACTCTCTGTGGTG
Il5	mouse	RT-qPCR	TGGAGATTCCCATGAGCACAG	GTAGGGACAGGAAGCCTCATC
Il9	mouse	RT-qPCR	TGCTCTTCAGTTCTGTGCTGG	GACGGAGAGACACAAGCAGC
Lgr5	mouse	RT-qPCR	CCTACTCGAAGACTTACCCAGT	GCATTGGGGTGAATGATAGCA
Lyz1	mouse	RT-qPCR	CTGTGGGATCAATTGCAGTG	GAATGCCTTGGGGATCTCTC
Muc2	mouse	RT-qPCR	ATGCCCACCTCCTCAAAGAC	GTAGTTTCCGTTGGAACAGTGAA
Muc3	mouse	RT-qPCR	AGGAGGCTGGAGAGGACTTTG	GCTGACATTTGCCGTAGCTGC
Pou2f3	mouse	RT-qPCR	CCCATGCACACAGAGATCAA	GCCATTTCGATCATTTCCTG
Retnlb	mouse	RT-qPCR	ATCAAGGAAGCTCTCAGTCG	CCACAAGCACATCCAGTGAC
Sirt6	mouse	RT-qPCR	ACGTCAGAGACACGGTTGTG	CCTCTACAGGCCCGAAGTC
Slc5a1	mouse	RT-qPCR	GTGGTACCGTTGGAGGCTT	CCACAAAGTGACCACTTCCA
Socs3	mouse	RT-qPCR	GAAGATTCCGCTGGTACTGAG	GCTGGGTCACTTTCTCATAGG
Stat6	mouse	RT-qPCR	TGTCCTGGACCTCACCAAAC	TCAGAGTCGCTAAAGCGGAG
Tff3	mouse	RT-qPCR	TCCAAGCCAATGTATGGTGC	TGGGATACTGGAGTCAAAGC
Trpm5	mouse	RT-qPCR	ATCTTTGGGCAAATCCCTCT	AGAGATTAGGGCAGGAAGCC
SOCS3	human	RT-qPCR	ATGAGAACTGCCAGGGAATC	TCTCTCTTCCACCTTTCCCAG
ACTB-ChIP	human	ChIP	TGCAGGAAAACTGGAATCCT	GGACCCGACCACAAACTTAG
SOCS3-ChIP-1	human	ChIP	CGCCTCTGCCAGAAATCAGC	GAGACAAAGCGCGACGAGAG
SOCS3-ChIP-2	human	ChIP	TGTGGGCCATCTGGCAGTAG	GATCTGCGGGAATGGCTGTC
SOCS3-ChIP-3	human	ChIP	TCAAGTGATCCTCTCGCCTCC	GGGACTCAGTGAGATTTGGAC

Supplementary Table 2. The information of the primary antibodies used in this study

Antibody	Catalog number	Vendor	Application	Dilution
Actinin	11313-2-AP	Proteintech	WB	1:10000
DCLK1	ab109029	Abcam	IHC	1:100
FLAG	F1804	Sigma-Aldrich	WB&ChIP	1:5000 (WB)<br>1:50 (ChIP)
Histone H3 (acetyl K56)	A7256	Abclonal	ChIP	1:20
Ki67	ab16667	Abcam	IHC	1:200
MYC tag	60003-2-Ig	Proteintech	WB	1:1000
MUC2	sc-15334	Santa Cruz	IF	1:200
SOCS3	MAB5696-SP	R&D Systems	WB	1:2500
SIRT6	12486	CST	WB	1:4000
SIRT6	ab62739	Abcam	IF	1:800
STAT6	51073-1-AP	Proteintech	WB	1:3000
Phospho-STAT6 (Y641)	ab263947	Abcam	WB, IF	1:500
α-Tubulin	11224-1-AP	Proteintech	WB	1:20000
CD3 FITC, clone 17A2	100203	BioLegend	Flow cytometry	1:100
CD4 PE/Cyanine7, clone GK1.5	100421	BioLegend	Flow cytometry	1:100
CD11b PE, clone M1/70	101207	BioLegend	Flow cytometry	1:100
CD11c PE, clone N418	117307	BioLegend	Flow cytometry	1:100
CD16/32, clone 93	101319	BioLegend	Flow cytometry	1:200
CD19 PE, clone 6D5	115507	BioLegend	Flow cytometry	1:100
CD45 BV510, clone 30-F11	103137	BioLegend	Flow cytometry	1:100
CD45R/B220 PE, clone RA3-6B2	103207	BioLegend	Flow cytometry	1:100
CD49b PE, clone HMα2	103506	BioLegend	Flow cytometry	1:400
CD90.2 PerCP, clone 30-H12	105321	BioLegend	Flow cytometry	1:50
IL-17RB APC, clone 9B10	146307	BioLegend	Flow cytometry	1:50
Ly-6G/Ly-6C (Gr-1) PE, clone RB6-8C5	108407	BioLegend	Flow cytometry	1:100
HRP-Goat Anti-Mouse IgG (H+L)	SA00001-1	Proteintech	WB	1:5000
HRP-Goat Anti-Rabbit IgG (H+L)	SA00001-2	Proteintech	WB	1:5000
Cy3-labeled Goat Anti-Rabbit IgG (H+L)	A0516	Beyotime	IF	1:500
