# Supplementary material for: Sirtuin 6 maintains epithelial STAT6 activity to support intestinal tuft cell development and type 2 immunity
Source: Nat Commun. 2022 Sep 3;13:5192. doi: 10.1038/s41467-022-32846-4 (PMC9440928; doi:10.1038/s41467-022-32846-4)
Supplement: Supplementary file 3 — Reporting Summary [file 41467_2022_32846_MOESM3_ESM.pdf]

nature portfolio

Corresponding author(s): Xiwen Xiong
Qingzhi Wang

Last updated by author(s): Aug 11, 2022

Reporting Summary

Nature Portfolio wishes to improve the reproducibility of the work that we publish. This form provides structure for consistency and transparency in reporting. For further information on Nature Portfolio policies, see our Editorial Policies and the Editorial Policy Checklist.

Statistics

For all statistical analyses, confirm that the following items are present in the figure legend, table legend, main text, or Methods section.

n/a	Confirmed	
☐	☒	The exact sample size (n) for each experimental group/condition, given as a discrete number and unit of measurement
☐	☒	A statement on whether measurements were taken from distinct samples or whether the same sample was measured repeatedly
☐	☒	The statistical test(s) used AND whether they are one- or two-sided<br>*Only common tests should be described solely by name; describe more complex techniques in the Methods section.*
☒	☐	A description of all covariates tested
☐	☒	A description of any assumptions or corrections, such as tests of normality and adjustment for multiple comparisons
☐	☒	A full description of the statistical parameters including central tendency (e.g. means) or other basic estimates (e.g. regression coefficient) AND variation (e.g. standard deviation) or associated estimates of uncertainty (e.g. confidence intervals)
☐	☒	For null hypothesis testing, the test statistic (e.g. F, t, r) with confidence intervals, effect sizes, degrees of freedom and P value noted<br>*Give P values as exact values whenever suitable.*
☒	☐	For Bayesian analysis, information on the choice of priors and Markov chain Monte Carlo settings
☒	☐	For hierarchical and complex designs, identification of the appropriate level for tests and full reporting of outcomes
☒	☐	Estimates of effect sizes (e.g. Cohen's d, Pearson's r), indicating how they were calculated

Our web collection on statistics for biologists contains articles on many of the points above.

Software and code

Policy information about availability of computer code

Data collection	H&E, IHC, IF images were obtained using ImageView.
Data analysis	Microsoft Excel 2021, Image J 1.50i, GraphPad Prism 8, FlowJo v10.6.2, Hisat2 2.1.0, HTSeq-count v.0.13.5, DEseq2 v1.30.1, GSEA 4.1.0

For manuscripts utilizing custom algorithms or software that are central to the research but not yet described in published literature, software must be made available to editors and reviewers. We strongly encourage code deposition in a community repository (e.g. GitHub). See the Nature Portfolio guidelines for submitting code & software for further information.

Data

Policy information about availability of data

All manuscripts must include a data availability statement. This statement should provide the following information, where applicable:

- Accession codes, unique identifiers, or web links for publicly available datasets
- A description of any restrictions on data availability
- For clinical datasets or third party data, please ensure that the statement adheres to our policy

RNA-sequencing data generated during this study have been deposited in the Gene Expression Omnibus (GEO) under accession number GSE202470 (https://www.ncbi.nlm.nih.gov/geo/query/acc.cgi?acc=GSE202470). The authors declare that all other data supporting the findings of this study are available from the corresponding author upon reasonable request. Source data are provided with this paper.

Field-specific reporting

Please select the one below that is the best fit for your research. If you are not sure, read the appropriate sections before making your selection.

- [x] Life sciences
- [ ] Behavioural & social sciences
- [ ] Ecological, evolutionary & environmental sciences

For a reference copy of the document with all sections, see nature.com/documents/nr-reporting-summary-flat.pdf

Life sciences study design

All studies must disclose on these points even when the disclosure is negative.

Sample size	No statistical methods were used to predetermine sample size due to the nature of the study. Sample size determination is based on the previous experience to obtain significance and reproducibility. Basically, the sample size follows common standards employing three or more biological replicates. All sample sizes are listed in the corresponding figure legends or on the figures.
Data exclusions	No data exclusions.
Replication	All experiments were conducted at least two times independently, and similar results were adopted for further analysis to guarantee reproducibility.
Randomization	Samples were randomly allocated into the study.
Blinding	This study included a lot of complicated experimental design and animal experiments, the feasibility of blinding was poor, thus blinding was not efficiently applied. The investigators were blinded to group allocation during data collection and analysis.

Reporting for specific materials, systems and methods

We require information from authors about some types of materials, experimental systems and methods used in many studies. Here, indicate whether each material, system or method listed is relevant to your study. If you are not sure if a list item applies to your research, read the appropriate section before selecting a response.

Materials & experimental systems

n/a	Involved in the study	
[ ]	[x]	Antibodies
[ ]	[x]	Eukaryotic cell lines
[x]	[ ]	Palaeontology and archaeology
[ ]	[x]	Animals and other organisms
[x]	[ ]	Human research participants
[x]	[ ]	Clinical data
[x]	[ ]	Dual use research of concern

Methods

n/a	Involved in the study	
[x]	[ ]	ChIP-seq
[ ]	[x]	Flow cytometry
[x]	[ ]	MRI-based neuroimaging

Antibodies

Antibodies used

Primary antibody:
Actinin Cat: 11313-2-AP Proteintech WB 1:10000
DCLK1, clone EPR6085 Cat: ab109029 Abcam IHC 1:100
FLAG, clone M2 Cat: F1804 Sigma-Aldrich ChIP 1:50, WB 1:5000
Histone H3 (acetyl K56) Cat: A7256 Abclonal ChIP 1:20
Ki67, clone SP6 Cat: ab16667 Abcam IHC 1:200
MYC tag, clone 1A5A2 Cat: 60003-2-Ig Proteintech WB 1:1000
MUC2 Cat: sc-15334 Santa Cruz IF 1:200
SOCS3, clone 516919 Cat: MAB5696-SP R&D Systems WB 1:2500
SIRT6, clone D8D12 Cat: 12486 CST WB 1:4000
SIRT6 Cat: ab62739 Abcam IF 1:800
STAT6 Cat: 51073-1-AP Proteintech WB 1:3000
Phospho-STAT6 (Y641) Cat: ab263947 Abcam WB IF 1:500
α-Tubulin Cat: 11224-1-AP Proteintech WB 1:20000
CD3 FITC, clone 17A2 Cat: 100203 BioLegend Flow cytometry 1:100
CD4 PE/Cyanine7, clone GK1.5 Cat: 100421 BioLegend Flow cytometry 1:100
CD11b PE, clone M1/70 Cat: 101207 BioLegend Flow cytometry 1:100
CD11c PE, clone N418 Cat: 117307 BioLegend Flow cytometry 1:100
CD16/32, clone 93 Cat: 101319 BioLegend Flow cytometry 1:200

	CD19 PE, clone 6D5 Cat: 115507 BioLegend Flow cytometry 1:100<br>CD45 BV510, clone 30-F11 Cat: 103137 BioLegend Flow cytometry 1:100<br>CD45R/B220 PE, clone RA3-6B2 Cat: 103207 BioLegend Flow cytometry 1:100<br>CD49b PE, clone HMα2 Cat: 103506 BioLegend Flow cytometry 1:400<br>CD90.2 PerCP, clone 30-H12 Cat: 105321 BioLegend Flow cytometry 1:50<br>IL-17RB APC, clone 9B10 Cat: 146307 BioLegend Flow cytometry 1:50<br>Ly-6G/Ly-6C (Gr-1) PE, clone RB6-8C5 Cat: 108407 BioLegend Flow cytometry 1:100<br><br>Secondary antibody:<br>HRP-Goat Anti-Mouse IgG (H+L) Cat: SA00001-1 Proteintech WB 1:5000<br>HRP-Goat Anti-Rabbit IgG (H+L) Cat: SA00001-2 Proteintech WB 1:5000<br>Cy3-labeled Goat Anti-Rabbit IgG (H+L) Cat: A0516 Beyotime IF 1:500
Validation	Validation of the use of Actinin antibody for mouse in WB has been provided by the manufacture's website. https://www.ptgcn.com/products/ACTN1-Antibody-11313-2-AP.htm<br><br>Validation of the use of DCLK1 antibody for mouse in IHC has been provided by the manufacture's website. https://www.abcam.com/dcamkl1-antibody-epr6085-ab109029.html<br><br>Validation of the use of FLAG antibody for mouse in WB and ChIP has been provided by the manufacture's website. https://www.sigmaaldrich.cn/CN/zh/product/sigma/f1804<br><br>Validation of the use of Histone H3 (acetyl K56) antibody for mouse in ChIP has been provided by the manufacture's website. https://abclonal.com.cn/catalog/A7256<br><br>Validation of the use of Ki67 antibody for mouse in IHC has been provided by the manufacture's website. https://www.abcam.com/ki67-antibody-sp6-ab16667.html<br><br>Validation of the use of MYC tag antibody in WB has been provided by the manufacture's website. https://www.ptgcn.com/products/MYC-Antibody-60003-2-Ig.htm<br><br>Validation of the use of SOCS3 antibody for mouse and human in WB has been provided by the manufacture's website. https://www.rndsystems.com/cn/products/human-mouse-socs-3-antibody-516919_mab5696<br><br>Validation of the use of MUC2 antibody for mouse in IHC has been provided by the manufacture's website. https://www.scbt.com/zh/p/mucin-2-antibody-h-300?requestFrom=search<br><br>Validation of the use of SIRT6 antibody for mouse and human in WB has been provided by the manufacture's website. https://www.cellsignal.cn/products/primary-antibodies/sirt6-d8d12-rabbit-mab/12486?site-search-type=Products&N=4294956287&Ntt=12486&fromPage=plp&_requestid=3909510<br><br>Validation of the use of SIRT6 antibody for mouse in IF has been provided by the manufacture's website (https://www.abcam.com/sirt6-antibody-ab62739.html) and by Supplementary Figure 5a in our article.<br><br>Validation of the use of STAT6 antibody for mouse and human in WB has been provided by the manufacture's website. https://www.ptgcn.com/products/STAT6-Antibody-51073-1-AP.htm<br><br>Validation of the use of Phospho-STAT6 (Y641) antibody for mouse and human in WB and IF has been provided by the manufacture's website (https://www.abcam.com/stat6-phospho-y641-antibody-epr22599-78-ab263947.html) and by Supplementary Figure 5b in our article.<br><br>Validation of the use of α-Tubulin antibody for mouse and human in WB has been provided by the manufacture's website. https://www.ptgcn.com/products/TUBA1B-Antibody-11224-1-AP.htm<br><br>Validation of the use of CD3 FITC antibody for mouse in flow cytometry has been provided by the manufacture's website. https://www.biolegend.com/en-us/products/fitc-anti-mouse-cd3-antibody-45<br><br>Validation of the use of CD4 PE/Cyanine7 antibody for mouse in flow cytometry has been provided by the manufacture's website. https://www.biolegend.com/en-us/products/pe-cyanine7-anti-mouse-cd4-antibody-1919<br><br>Validation of the use of CD11b PE antibody for mouse in flow cytometry has been provided by the manufacture's website. https://www.biolegend.com/en-us/products/pe-anti-mouse-human-cd11b-antibody-349<br><br>Validation of the use of CD11c PE antibody for mouse in flow cytometry has been provided by the manufacture's website. https://www.biolegend.com/en-us/products/pe-anti-mouse-cd11c-antibody-1816<br><br>Validation of the use of CD16/32 antibody for mouse in flow cytometry has been provided by the manufacture's website. https://www.biolegend.com/en-us/products/trustain-fcx-anti-mouse-cd16-32-antibody-5683<br><br>Validation of the use of CD19 PE antibody for mouse in flow cytometry has been provided by the manufacture's website. https://www.biolegend.com/en-us/products/pe-anti-mouse-cd19-antibody-1530

Validation of the use of CD45 BV510 antibody for mouse in flow cytometry has been provided by the manufacture's website. https://www.biolegend.com/en-us/products/brilliant-violet-510-anti-mouse-cd45-antibody-7995

Validation of the use of CD45R/B220 PE antibody for mouse in flow cytometry has been provided by the manufacture's website. https://www.biolegend.com/en-us/products/pe-anti-mouse-human-cd45r-b220-antibody-447

Validation of the use of CD49b PE antibody for mouse in flow cytometry has been provided by the manufacture's website. https://www.biolegend.com/en-us/products/pe-anti-mouse-cd49b-antibody-299

Validation of the use of CD90.2 PerCP antibody for mouse in flow cytometry has been provided by the manufacture's website. https://www.biolegend.com/en-us/products/percp-anti-mouse-cd90-2-thy1-2-antibody-4278

Validation of the use of IL-17RB APC antibody for mouse in flow cytometry has been provided by the manufacture's website. https://www.biolegend.com/en-us/products/apc-anti-mouse-il-17rb-antibody-10459

Validation of the use of Ly-6G/Ly-6C (Gr-1) PE antibody for mouse in flow cytometry has been provided by the manufacture's website. https://www.biolegend.com/en-us/products/pe-anti-mouse-ly-6g-ly-6c-gr-1-antibody-460

Validation of the use of HRP-Goat Anti-Mouse lgG (H+L) antibody in WB has been provided by the manufacture's website. https://www.ptgcn.com/products/HRP-conjugated-Affinipure-Goat-Anti-Mouse-IgG-H-L-secondary-antibody.htm

Validation of the use of HRP-Goat Anti-Rabbit IgG (H+L) antibody in WB has been provided by the manufacture's website. https://www.ptgcn.com/products/HRP-conjugated-Affinipure-Goat-Anti-Rabbit-IgG-H-L-secondary-antibody.htm

Validation of the use of Cy3-labeled Goat Anti-Rabbit IgG (H+L) antibody in WB has been provided by the manufacture's website. https://www.beyotime.com/product/A0516.htm

Eukaryotic cell lines

Policy information about cell lines

Cell line source(s)	293T cells, National Collection of Authenticated Cell Cultures (NCACC, Shanghai, China);<br>NCM460 cells, LONZA.
Authentication	All cell lines were routinely authenticated in our lab by morphological examination using microscopy.
Mycoplasma contamination	Negative for mycoplasma.
Commonly misidentified lines<br>(See ICLAC register)	No commonly misidentified cell lines were used.

Animals and other organisms

Policy information about studies involving animals; ARRIVE guidelines recommended for reporting animal research

Laboratory animals	Mouse strains used in this study:<br>Sirt6 flox/flox, C57BL/6J Background. (Cell Metab,12(3):224-36.)<br>TgSIRT6 flox/flox, C57BL/6J Background. (J Hepatol,71(5):960-969.)<br>Villin-Cre, C57BL/6J Background. (Genesis, 39(3):186-93.)<br>TgSTAT6vt, C57BL/6J Background. (Generated by GemPharmatech, Nanjing, China)<br>wild-type C57BL/6J. (Purchased from GemPharmatech, Nanjing, China)<br>Both male and female mice with age from 2-4 months were used in this study.
Wild animals	The study did not involve wild animals.
Field-collected samples	The study did not involve field-collected samples.
Ethics oversight	Xinxiang Medical University, IACUC

Note that full information on the approval of the study protocol must also be provided in the manuscript.

Flow Cytometry

Plots

Confirm that:

- [x] The axis labels state the marker and fluorochrome used (e.g. CD4-FITC).
- [x] The axis scales are clearly visible. Include numbers along axes only for bottom left plot of group (a 'group' is an analysis of identical markers).
- [x] All plots are contour plots with outliers or pseudocolor plots.
- [x] A numerical value for number of cells or percentage (with statistics) is provided.

Methodology

Sample preparation	Small intestine was flushed, opened longitudinally, and cut into ~5-6 pieces. DTT and EDTA solutions were used sequentially to remove intra-epithelial lymphocytes and epithelial cells. The remaining tissue was washed and digested with collagenase for 45 minutes at 37 °C with shaking. Lamina propria (LP) lymphocytes were pelleted after filtering the digested tissue through 100 μm filters. Cells were fixed and then stained with relevant antibodies at 4 °C for 30 min.
Instrument	CytoFLEX (Beckman Coulter)
Software	FlowJo (v10.6.2)
Cell population abundance	Cell sorting not employed.
Gating strategy	Based on the pattern of FSC-A/SSC-A, the debris were excluded. Singlets were gated according to the pattern of FSC-W vs. FSC-H, then SSC-W vs. SSC-H. Single cells were then selected for viable, lineage negative (CD19-CD11b-CD49b-CD11c-Gr-1-B220-), CD45+ cells. Within the CD3-CD4- subset, ILC2 population is positive for CD90 and IL-17RB.

- [x] Tick this box to confirm that a figure exemplifying the gating strategy is provided in the Supplementary Information.
